# Extrachromosomal Circular DNA (eccDNA): From Chaos to Function

**DOI:** 10.3389/fcell.2021.792555

**Published:** 2022-01-06

**Authors:** Shanru Zuo, Yihu Yi, Chen Wang, Xueguang Li, Mingqing Zhou, Qiyao Peng, Junhua Zhou, Yide Yang, Quanyuan He

**Affiliations:** ^1^ Department of Pharmacy, The Third Xiangya Hospital, Central South University, Changsha, China; ^2^ The Key Laboratory of Model Animals and Stem Cell Biology in Hunan Province, School of Medicine, Hunan Normal University, Changsha, China; ^3^ Department of Orthopaedics, Wuhan Union Hospital, Wuhan, China; ^4^ Department of Obstetrics and Gynecology, The Third Xiangya Hospital of Central South University, Changsha, China; ^5^ Zhongshan Hospital Affiliated to Sun Yat-Sen University, Zhongshan People’s Hospital, Zhongshan, China; ^6^ Institute of Chinese Medicine, Hunan Academy of Traditional Chinese Medicine and Innovation Centre for Science and Technology, Hunan University of Chinese Medicine, Changsa, China; ^7^ Chongqing Key Laboratory for Pharmaceutical Metabolism Research, College of Pharmacy, College of Traditional Chinese Medicine, Chongqing Medical University, Chongqing, China

**Keywords:** eccDNA, circulome, biogenesis, cancer, biomarker

## Abstract

Extrachromosomal circular DNA (eccDNA) is a type of double-stranded circular DNA that is derived and free from chromosomes. It has a strong heterogeneity in sequence, length, and origin and has been identified in both normal and cancer cells. Although many studies suggested its potential roles in various physiological and pathological procedures including aging, telomere and rDNA maintenance, drug resistance, and tumorigenesis, the functional relevance of eccDNA remains to be elucidated. Recently, due to technological advancements, accumulated evidence highlighted that eccDNA plays an important role in cancers by regulating the expression of oncogenes, chromosome accessibility, genome replication, immune response, and cellular communications. Here, we review the features, biogenesis, physiological functions, potential functions in cancer, and research methods of eccDNAs with a focus on some open problems in the field and provide a perspective on how eccDNAs evolve specific functions out of the chaos in cells.

## Introduction

**FIGURE 1:**
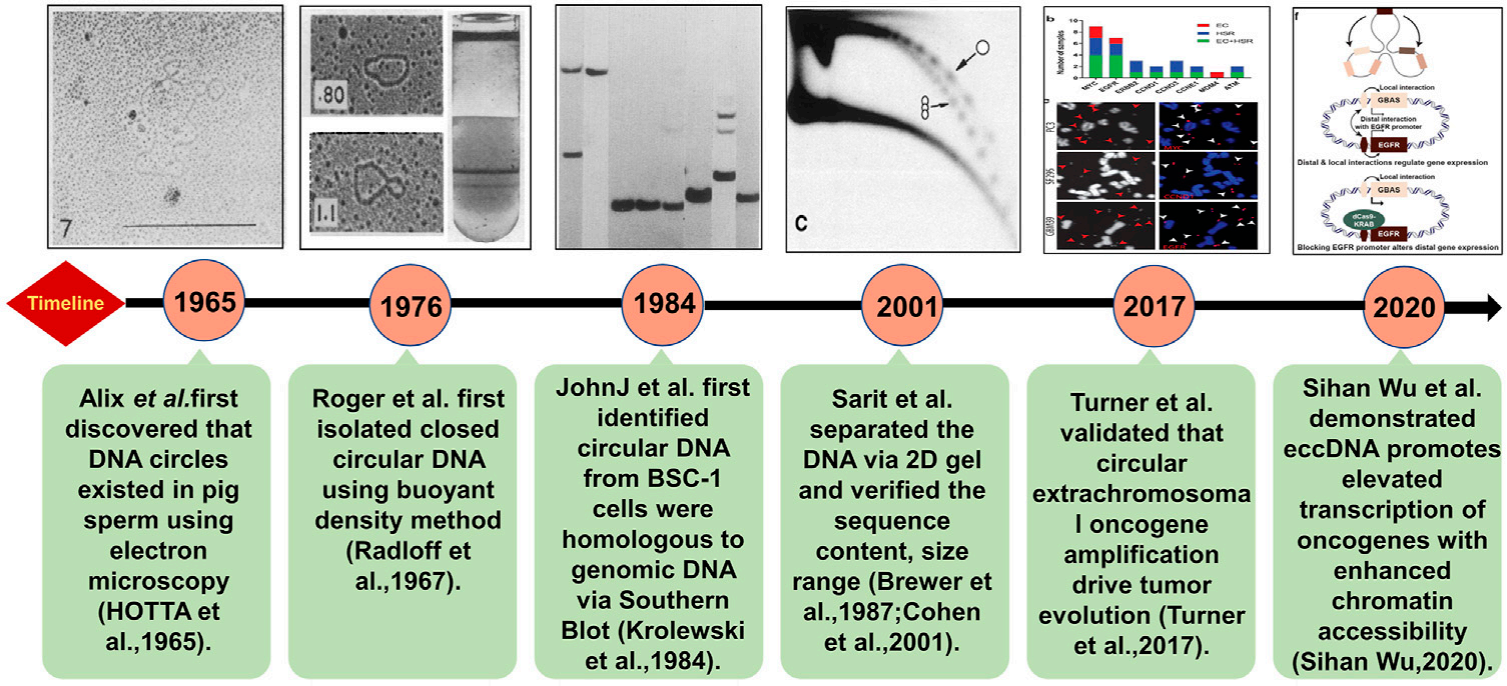
Brief discovery history of eccDNA. The technologies and key discoveries were illustrated following the timeline of the milestone studies. The detailed descriptions are as following: in 1965, Alix Bassel and Yasuo Hoota first observed eccDNAs in boar sperm under the electron microscope (Hotta and Bassel, 1965). The Roger’s group extracted eccDNA in HeLa cells by CsCl gradient purification in 1976 (Radloff et al., 1967). In 1984, Krolewski found that eccDNA molecules were homologous to genomic DNA (Krolewski et al., 1984) using Southern blot indicating that eccDNAs may be derived from chromosomes. Neutral–neutral two-dimensional (2D) gel electrophoresis combined with standard procedures of Southern blots and hydridization verified useful for characterizing eccDNA including its size range and sequence content, and it can prove eccDNA organized as discrete multimers (Brewer and Fangman, 1987; Cohen and Mechali, 2001). With rapid advances in high-throughput DNA sequencing, more eccDNAs have been identified and quantified on a global level (Turner et al., 2017). Furthermore, the architecture of eccDNA can be directly observed by super resolution (SR) confocal microscopy (Sihan Wu, 2020).

EccDNA refers to a type of double-stranded circular DNA that is derived and free from chromosomes. In 1965, Alix Bassel and Yasuo Hoota first observed eccDNA in boar sperm using the electron microscope (Hotta and Bassel, 1965). From there, many efforts have been taken to figure out the features and functions of eccDNAs (Figure 1). It is clear now that eccDNA is ubiquitous in eukaryotic cells and has been identified in yeasts, plants, *Oxytricha*, *Xenopus*, pigeons, and human cells. (Cohen and Méchali, 2002; Møller et al., 2015; Hull et al., 2019; Yerlici et al., 2019; Molin et al., 2020a, Molin et al., 2020b; Møller et al., 2020; Sin et al., 2021; Wang K et al., 2021; Zhu et al., 2021). It can be derived from everywhere in a genome with sizes ranging from hundreds of base pairs (bp) to several mega bases (Mb). According to the size and origin, eccDNAs can be categorized into mitochondria DNAs (mtDNAs), episomes, double minutes (DMs) (100kb∼3 Mb), telomeric circle (t-circles), small polydispersed circular DNA (spcDNA) (100bp∼10 kb), and microDNA (100–400 bp) (Wang M et al., 2021).Some studies revealed that episomes can be polymerized into DMs in cancer cells suggesting the possibility that one type of eccDNA can be transformed to others by polymerization or fragmentation with subsequent recircularization (Wahl et al., 1984; Carroll et al., 1988).

As the counts, compositions, origin, and expression patterns of eccDNA are dramatically dynamic and diverse in cells and tissues, characterizing the functions and underline mechanisms of eccDNAs are extremely challenging (Turner et al., 2017). Many fundamental open questions about eccDNAs remained unanswered. For example, is eccDNA a byproduct or essential player of cellular procedures? Do the sequences of eccDNAs matter for their functions? What is the role of eccDNAs in cancer initiation and progression? et al. Some excellent reviews (Cao et al., 2021; Ling et al., 2021; Wang T et al., 2021) have summarized the characteristics and functions of eccDNA. Here, we review the last progress in the field and try to find the clues to answer the above questions.

## The Biogenesis of ECCDNA

**FIGURE 2:**
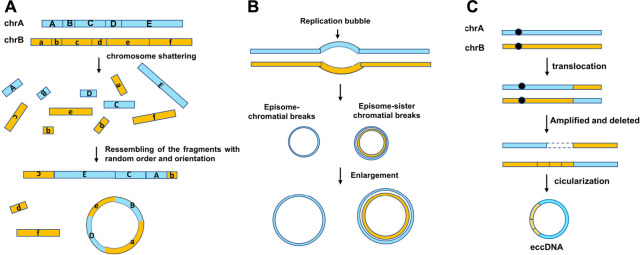
Three mechanisms of eccDNA biogenesis. **(A)** “Chromothripsis” model, chromothripsis is the generation of DNA double-strand breaks. Fragments are joined together in a random order and orientation by DNA repair machineries. The new chromosomes may contain complex structural rearrangements and structural variants. **(B)** Episome model, DNA bidirectionally replicates leading to two replication forks, and the region between them is the replication bubble. The replication fork collapse when the error in replication take place, and then, the replication bubbles drop and form shape of an episome. They can enlarge to form eccDNAs by replication and recombination. **(C)** Translocation–excision–deletion–amplification model. Gene translocation occurs near the chromosome. The fragment next to the translocation positions can be amplified, deleted, and circularized, generating the genesis of eccDNA.

Several models of the eccDNA biogenesis have been proposed: 1) The chromothripsis model (Figure 2A): Chromothripsis is the catastrophic shattering of a chromosome followed by massive genomic rearrangement in a random order, leading to complex genomic structural rearrangements in confined genomic regions. As the “shattering” procedure results in clustered DNA double-strand breaks (DSBs), following by DNA repairing or aberrant DNA replication, and which make it a perfect circumstance to generate eccDNA (Nazaryan-Petersen et al., 2020); in 2021, Wang’s team (Wang Y et al., 2021) reported that apoptosis can promote eccDNA generation in human and mouse cells. It is not surprising; apoptosis, just like chromothripsis, can induce DNA fragmentation and may provide massive DNA fragments for eccDNA formation; 2) Episome model (Figure 2B). Replication fork stalling may result in replication fork collapse, and the replication bubble subsequently falls off the chromosome, transforms into an extrachromosomal circular molecule named as episome (Gu et al., 2020); 3) Translocation–excision–deletion–amplification model (Figure 2C) (Boon et al., 2001; Van Roy et al., 2006). In this model, DNA rearrangements take place close to translocation sites on the chromosome. The fragment in proximity to the translocation breakpoints can be amplified, deleted, and circularized, resulting in the accumulation of eccDNAs.

The above studies indicated that the DNA damage repairing pathways including homologous recombination (HR) or nonhomologous end-joining (NHEJ) and microhomology-mediated end-joining (MMEJ) are involved in the formation of microDNAs (Dillon et al., 2015; Paulsen et al., 2021). The cells lack 53BP1 and XRCC4 promoting the canonical-NHEJ (c-NHEJ), resulting in more microDNA. On the contrary, the deficiency of NBS1, POLQ, RAD54, MLH1, MSH2, MSH3, FAN1, and NBS results in the decrease of microDNA (Paulsen et al., 2021). The short, reverted repeats at both ends of junction sites provide perfect places to initialize homologous recombination (HR) during eccDNA formation. Furthermore, some key players in NHEJ and MMEJ pathways, such as DNA ligases IV and III, were found to contribute to the eccDNA generation (Cohen et al., 2006; Wang K et al., 2021). Additionally, DNA replication and transcription were also proposed as the mechanisms underlying the formation of eccDNAs. For instance, the most transcribed protein-coding gene in the muscle, titin (TTN), has the greatest amount of eccDNAs (Møller et al., 2018). On the contrary, noncoding gene areas show a lower correlation with the eccDNA frequency (Dillon et al., 2015; Møller et al., 2018).

**FIGURE 3:**
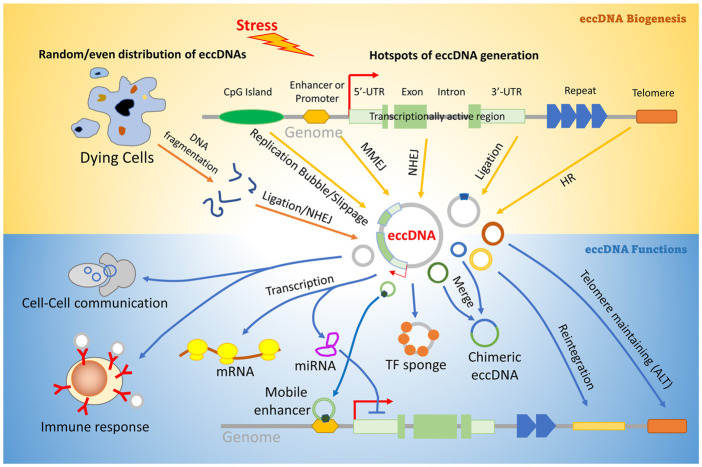
Pathways involved in eccDNA biogenesis and function. Stress may induce aberrant DNA replication, transcription, or even genomic DNA broken into random fragments, and generate linear DNA fragments. Some of them are ligated and circularized by DNA damage repair pathways including HR (homologous recombination), NHEJ (Non-homologous end joining), Ligation by DNA ligases IV and III, and MMEJ (microhomology mediated end-joining). EccDNAs can regulate many downstream biological procedures including transcription, telomere protection, gene translocation, immune response, and cell–cell communication. Description about details can be found in the text.

It is widely accepted that eccDNA origination is not restricted to a particular locus in the genome (Møller et al., 2020; Wang T et al., 2021). However, the pattern of eccDNA distribution is still controversial. Some studies reported that the vast majority of eccDNA originates from repetitive elements (Motejlek et al., 1993; Cohen and Mechali, 2001; Moller et al., 2015); others suggested that they prefer to reside in some hotspots such as UTRs of genes, GC islands, and transcriptionally activated chromatins (Shibata et al., 2012; Mehanna et al., 2017). A recent study supported an even distribution model in drug-induced apoptosis cells (Wang Y et al., 2021). Although the driving force of these patterns is still unknown, it is reasonable to speculate that eccDNA origination may be determined by the mechanisms of eccDNA generation under different cellular conditions (Figure 3).

## The Amplification of eccDNA

Most of eccDNA will be degraded rapidly except the ones who can amplify themselves. However, what are the mechanisms of eccDNA amplification is controversial. Some studies suggested that eccDNA amplification relies on DNA replication and mitosis (Baker and Grant, 2007; Herrup and Yang, 2007); others have found that the eccDNA level increases when the ongoing replication is blocked by inhibitors (Sunnerhagen et al., 1986) or even in the absence of any DNA replication (Cohen et al., 2009). However, it is not clear whether these eccDNA levels increasing are a result from amplification or higher levels of biogenesis. We speculate that eccDNAs can be amplified in a way independent of mitosis because many of them don’t have replication origins which are required by regular DNA replication.

## Regulatory Mechanisms of eccDNA

Accumulated evidence suggested that eccDNA may regulate many cellular procedures (see the function sections below) through distinct mechanisms (Figure 3): 1) Biogenesis of eccDNAs containing whole gene may lead to a gain in the gene copy number and enhance the transcription of the gene (Turner et al., 2017); 2) Forming R-loop by hybridization with mRNA to regulate its translational efficiency (Wiedemann et al., 2016); 3) Titrating the components of the replication or transcription machinery and result in an inability to replicate or transcribe genomic DNA, which eventually induces cell growth arrest and death; (Paulsen et al., 2019; Zhu, et al., 2021); 4) Acting as trans-acting factors (such as super enhancer) to regulate the gene expression by regulating the epigenetic statue and/or accessibility of the targeting gene (Wu et al., 2019); 5) Function as a cytokine to stimulate immune responses or mediate the intercellular communication (Poirier et al., 2018); 6. Genetic rearrangements by the reinsertion of eccDNA into the genome DNA (Pavri, 2017); 7. Protecting the telomeres in ALT cells (McEachern, 2000; Cech, 2004).

## Physiological Functions of eccDNA

It is already known that eccDNA plays an important role in a variety of physiological procedures including the following: 1) Stress resistance and evolution. In plants, eccDNA plays a role to amplify and transmit the herbicide resistance gene in crop weeds which result in rapid glyphosate resistance and adaptive evolution (Koo et al., 2018). Furthermore, eccDNA was identified in the *C.elegans* germ line and may serve as genetic materials to be inherited by the offspring (Shoura et al., 2017); 2) Functional enhancement. In humans, the most transcribed protein-coding gene in the muscle, titin (TTN), has the greatest amount of eccDNAs (Møller et al., 2018), and suggesting eccDNA may help host cell fulfill their function. 3) Aging. EccDNA has been found accumulated in old cells and inducing aging in yeast (Hull et al., 2019). It was also amplified during aging in senescence-resistant SAM-R mice and associated with premature aging and shorter lifespan (Yamagishi et al., 1985). Accumulated results highlighted the proposed role of eccDNAs in age-related CNS diseases, but direct evidence is still absent (Shibata et al., 2012); 4) Genome stability maintenance. Certain types of eccDNAs were originated from telomeres **(**t-circles) and centromeres (major satellite repeats), that are fundamental structures to maintain genome integrity. Especially, t-circle is essential to maintain telomeres in ATL cells (Neumann et al., 2013); 5) Immune response. As not protected by chromatin, eccDNA may become an endogenous antigen to active autoimmune cells (Collins et al., 2004). Recently, the role of eccDNA in triggering innate immune response was uncovered (Wang M et al., 2021). Surprisingly, the study demonstrated that it is the circular nature but not the sequence of eccDNA critical to the immune response activation; 6) Cell-to-cell communications. The high stability, high mobility, and self-replication ability of eccDNA make it a perfect messenger to transduce and amplify signals among cells. Recent studies confirmed that eccDNA (especially microDNA) can be emitted and sensed by human and mouse cells efficiently *in vitro* (Wang T et al., 2021). In addition, the eccDNAs released by normal and tumor cells have been detected in the peripheral blood (Møller et al., 2018). These data suggested a potential role of eccDNA contributing to both local and long-range signal transduction between cells.

## Functions of eccDNA in Cancer

EcDNA refers to large eccDNA exclusively detected in tumors. Furthermore, DM is a specific type of ecDNA, which usually contains intact oncogenes and/or drug-resistance genes, which has been detected in most (182/200) kinds of human tumors (Fan et al., 2011). EcDNA, as the vehicles for oncogene and drug-resistance genes, enables them to be rapidly amplified, and lead to overexpression consequently (Turner et al., 2017). For instance, oncogenes EGFR and c-MYC were found in ecDNA and amplified in human cancer tissues than normal tissues (L'Abbate et al., 2014). In tumor cells from a SCLC (small cell lung cancer) patient who received methotrexate (MTX), a large quantity of DMs containing dihydrofolate reductase (DHFR), a drug-resistance gene, was amplified and overexpressed (Curt et al., 1983). Lacking a high-order chromatin structure, suppressing histone modifications, and insulator shackle make ecDNAs more accessible than their genome counterparts which may facilitate promoter–enhancer interactions, transcription initialization, and achieving additional expression (Morton et al., 2019). Furthermore, recent studies also suggested that ecDNA/DMs can function as mobile super-enhancers to enhance targeting genes transcription in genome-wide (Y. Zhu, et al., 2021). Additionally, the unbalanced segregation of ecDNA during mitotic divisions provides an additional layer of tumor heterogeneity which ultimately cause tumor adaptation to the microenvironment stresses and various therapies such as radiotherapy and chemotherapy. This may be the reason that tumors with ecDNA are frequently more aggressive, related to poorer survival outcomes (Bruckert et al., 2000).

The role of smaller eccDNAs (e.g., microDNAs) in cancer biology remains controversial. As they usually are too small to contain the full length of the gene (except for miRNA genes), whether its transcriptional products have any effects on cancer progression is unknown. However, they may be important for molecular sponging. As often originated from the 5′-UTRs of its parental gene, microDNA may provide additional binding sites for related transcription factors and therefore, acting as a sponge for transcription factors to control the gene expression and transcription homeostasis indirectly (Reon and Dutta, 2016; Paulsen et al., 2018). Additionally, Wang et al. reported that the microDNAs released from dying cells can dramatically induce type I interferon expression and innate immunostimulatory response. It is suspected that drug treatment on cancer cells may induce cell apoptosis with eccDNA generation and subsequent change in the tumor microenvironment dramatically. This work highlighted the potential role of microDNA in drug resistance of cancer cells and shed new light on cancer immunotherapy (Wang Y et al., 2021).

## Applications of eccDNA in Medicine

The entire set of circular DNA (Circulome) varies as a function of tissue type and health and disease conditions and can be used as a fingerprint of disease. Extracellular free eccDNAs are more stable than linear cell-free DNAs (cfDNAs) and have been detected in cancer tissues and peripheral blood of cancer patients (Kumar et al., 2017), suggesting the potential applying them as a novel type of biomarkers in liquid biopsy for the early detection of diseases, the monitoring of drug treatment response, and cancer survival.

Although many efforts have been made, the application of eccDNA in medicine is still at an early stage. In the mammalian repertoire of eccDNA, microDNAs may be the most promising biomarkers because they have smaller size, higher mobility, and higher abundance (account for up to 84% of the entire circulome) (Shibata et al., 2012; Dillon et al., 2015) than others. Furthermore, it exhibited lineage-specific or cell type-specific patterns in human cells (Hotta and Bassel, 1965; Dillon et al., 2015). However, the functions of microDNA are largely unknown, and very few of them are shared by patients, which raise the questions whether the sequence is a matter for them or not. Large cohort studies are needed to identify clinic-relevant microDNA patterns. Another potential eccDNA biomarker is mtDNA. Some studies have reported that the mtDNA copy number variation was correlated with cancer (Sozzi et al., 2003). For example, a high mtDNA copy number in peripheral blood leukocytes (PBLs) was associated with the increased risk of prostate cancer (PCa) and high tumor burden in PCa patients (Zhou et al., 2014). And paradoxically, another report indicated that low mtDNA abundance in PBLs correlated with aggressive PCa at diagnosis (Tu et al., 2015). Similarly, the mtDNA copy number was significantly lower in PBLs of patients with endometrial cancer than normal controls (Sun et al., 2016). As these associations between mtDNA amount and cancer risk are inconsistent and the underlying mechanisms are still unknown, huge gap needs to be filled before the clinical application of mtDNAs. Moreover, it remains unclear about eccDNAs’ presence in other bodily fluids such as saliva and urine. It is worthwhile to check the existence of eccDNAs in other bodily fluids.

## Advance of eccDNA Research Methods

**FIGURE 4:**
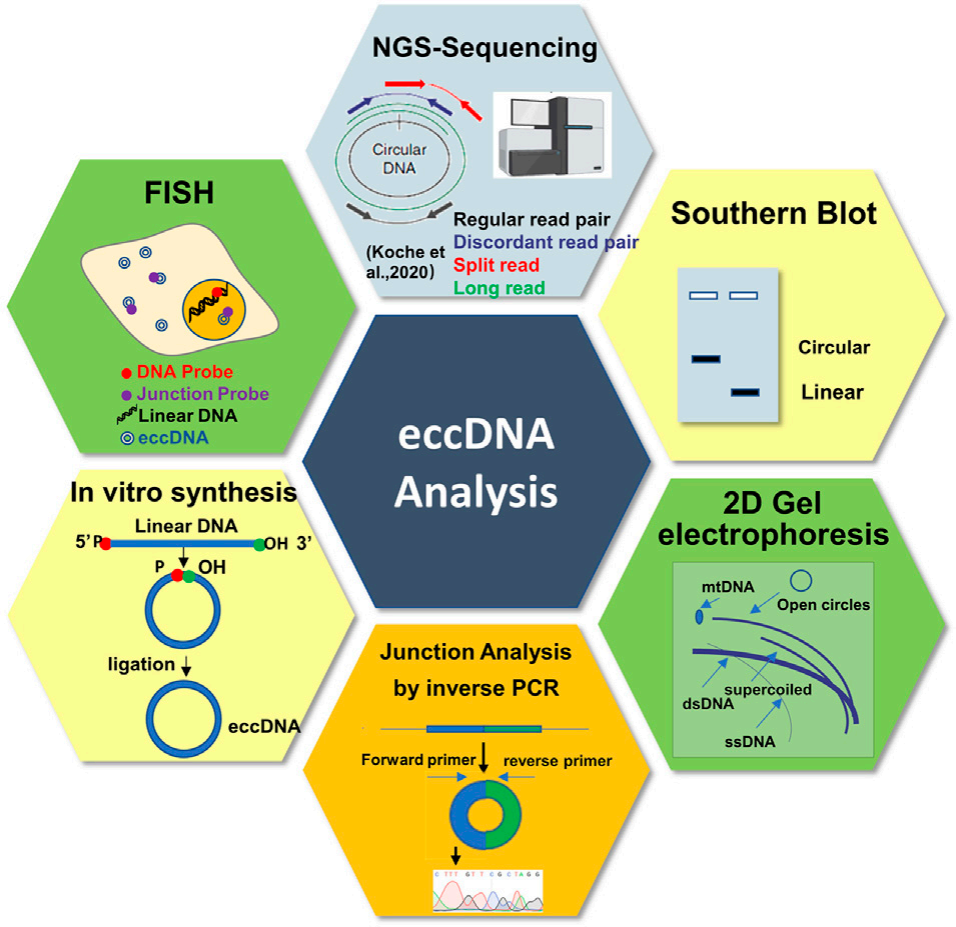
Schematic representation of the different approaches and tools to study circular DNA.

Many conventional methods were used to characterize the sequence, copy number, subcellular localization, and biological functions of eccDNAs (Figure 4), and which was reviewed by elsewhere (Wang T et al., 2021). However, these methods usually provide little information about eccDNA sequences and cannot provide the global view for the whole circulome. Currently, the next/third generation sequencing technologies (such as long-read sequencing (Nanopore and PacBio SMRT sequencing) and single cell sequencing (SC-Seq)) and the improved methods (such as Circle-seq, 4C-seq, PLAC-seq, and ATAC-seq) provide us revolutionary tools to investigate the circulome from various perspectives and with single base-pair and single-cell resolutions (Turner et al., 2017; Prada-Luengo et al., 2019; Rajkumar et al., 2019; Kumar et al., 2020; Mehta et al., 2020; Sin et al., 2020). Especially, the long-read sequencing coupled with the PCR-free eccDNA purification technology present a promising solution to overcome the limitation of short-read sequencing in detecting big eccDNAs and provide unbiased circulomics profiles (Wang M et al., 2021). Furthermore, we speculated that the SC-Seq may be the next dominant technology to decode the rule of eccDNA segregation to rebuild the evolutionary history of the tumor (van den Bos et al., 2018). Additionally, recently, many bioinformatics analysis tools and database for eccDNAs have been proposed (Rajkumar et al., 2019; Wu et al., 2019), which facilitate the rapid development of eccDNA research significantly. However, taking the extreme complexity of the circulome into consideration, the data about eccDNA are still very limited; intensive studies need to be carried out to accumulate and annotate eccDNAs in the future.

## Perspective: From Chaos to Function

For most of eccDNAs, the story may begin with a chaos result from certain internal and external stresses. The stress induces aberrant transcription, DNA replication, and recombination leading to the release of DNA fragments from some hotspots which were consequently recognized and “repaired” by DNA damage repairing mechanisms to form a circulated DNA. In dying or apoptosis cell, global genome DNA fragmentation generate a lot of DNA fragments in a random fashion; some of them were also “rescued” by DNA damage repairing mechanisms to form eccDNAs. We speculate that the former mechanism may majorly contribute to the formation of relatively longer eccDNAs (episomes and DMs), and the latter one is for shorter eccDNAs (spcDNA and microDNA). Most of the eccDNAs will be degraded rapidly, and only the ones who have the ability to amplify themselves and provide adaptive advantage to cells can survive. During the procedure of evolutionary selection, eccDNAs may migrate through cells to search for a good host. They can merge with others to create a new one and novel functions which may provide further advantages of the survival of themselves and hosts. Some eccDNAs may be even more “lucky” to get the chance to integrate into genomic DNA, which eventually fixes themselves in the cell’s genome. In tumors, this story may happen everywhere and all the time. EccDNAs help cancer cells develop drug resistance and escape from immune attack. Furthermore, it may also be possible that the eccDNAs originated from the cancer cell can transform target cells remotely through circulation and mediate the metastasis. In germ line, eccDNAs may serve as the secondary genetic materials and were passed to progeny and create additional genetic diversity in population. For somatic cells, too much random eccDNAs may be a burden. However, highly specific functional eccDNA may be a big help for them to fulfill their functions especially under stress conditions (Møller et al., 2018).

## Conclusion

The discovery of eccDNA may reshape our current understanding of the heredity, evolution, and diseases. EccDNA, as a new kind of genetic material, is mobile, flexible, functionable, tremendous diverse, and everywhere (Wang T et al., 2021). It lets all the cells different and makes quick adaptation and evolution possible. The biological significance of eccDNAs may be heavily underestimated. The eccDNA study is still in its infancy.

Future studies need to focus on 1) Figuring out the chromatin structural features and sequence characteristics of eccDNA on the circulome level; 2) Discovering key regulators and interaction partners of eccDNAs; 3) Exploring the eccDNA segregation procedure at the single cell level to understanding cancer development; 4) Applying it as a liquid biopsy biomarker to improve disease diagnosis, prognosis, and treatment selection; 5) Developing more convenience and sensitive detection methods and advanced tools to support eccDNA research. All these works will deepen our understanding of the nature of eccDNAs and open up new opportunities for cancer diagnosis and therapy.
